# Bone marrow stromal cells from multiple myeloma patients uniquely induce bortezomib resistant NF-κB activity in myeloma cells

**DOI:** 10.1186/1476-4598-9-176

**Published:** 2010-07-06

**Authors:** Stephanie Markovina, Natalie S Callander, Shelby L O'Connor, Guangwu Xu, Yufang Shi, Catherine P Leith, KyungMann Kim, Parul Trivedi, Jaehyup Kim, Peiman Hematti, Shigeki Miyamoto

**Affiliations:** 1 Program in Cellular and Molecular Biology and Medical Science Training Program, University of Wisconsin School of Medicine and Public Health, 750 Highland Avenue, Madison, Wisconsin, 53705, USA; 2 Department of Pharmacology, University of Wisconsin School of Medicine and Public Health, 750 Highland Avenue, Madison, Wisconsin, 53705, USA; 3 Section of Hematology/Oncology, Department of Medicine, University of Wisconsin School of Medicine and Public Health, 750 Highland Avenue, Madison, Wisconsin, 53705, USA; 4 University of Wisconsin Carbone Cancer Center, University of Wisconsin School of Medicine and Public Health, 750 Highland Avenue, Madison, Wisconsin, 53705, USA; 5 Department of Molecular Genetics, Microbiology and Immunology, University of Medicine and Dentistry of New Jersey, Robert Wood Johnson Medical School, 150 Bergen Street, Newark, New Jersey, 07103, USA; 6 University of Wisconsin Division of Hematopathology, Department of Pathology, University of Wisconsin School of Medicine and Public Health, 750 Highland Avenue, Madison, Wisconsin, 53705, USA; 7 Department of Biotatistics and Medical Informatics, University of Wisconsin School of Medicine and Public Health, 750 Highland Avenue, Madison, Wisconsin, 53705, USA

## Abstract

**Background:**

Components of the microenvironment such as bone marrow stromal cells (BMSCs) are well known to support multiple myeloma (MM) disease progression and resistance to chemotherapy including the proteasome inhibitor bortezomib. However, functional distinctions between BMSCs in MM patients and those in disease-free marrow are not completely understood. We and other investigators have recently reported that NF-κB activity in primary MM cells is largely resistant to the proteasome inhibitor bortezomib, and that further enhancement of NF-κB by BMSCs is similarly resistant to bortezomib and may mediate resistance to this therapy. The mediating factor(s) of this bortezomib-resistant NF-κB activity is induced by BMSCs is not currently understood.

**Results:**

Here we report that BMSCs specifically derived from MM patients are capable of further activating bortezomib-resistant NF-κB activity in MM cells. This induced activity is mediated by soluble proteinaceous factors secreted by MM BMSCs. Among the multiple factors evaluated, interleukin-8 was secreted by BMSCs from MM patients at significantly higher levels compared to those from non-MM sources, and we found that IL-8 contributes to BMSC-induced NF-κB activity.

**Conclusions:**

BMSCs from MM patients uniquely enhance constitutive NF-κB activity in MM cells via a proteinaceous secreted factor in part in conjunction with IL-8. Since NF-κB is known to potentiate MM cell survival and confer resistance to drugs including bortezomib, further identification of the NF-κB activating factors produced specifically by MM-derived BMSCs may provide a novel biomarker and/or drug target for the treatment of this commonly fatal disease.

## Background

The non-cancerous cells associated with tumors were recognized more than three decades ago to play considerable roles in tumor development, progression and maintenance [1]. Since then, there has been a significant effort to delineate the specific contributions of these cells to malignant cell behaviors. More specifically, numerous recent studies have found that these supportive cells, although not cancerous per se, are functionally and sometimes genetically different from their normal counterparts [1]. Studies done in the nineties like those by Cunha and colleagues in prostate cancer [2], and Bissell and colleagues in breast cancer [3] have helped to widen the focus of cancer researchers from just the tumor cells to considering also the components around them. As a result of this work, several cancer therapies have been engineered specifically to target interactions between tumor cells and their surrounding stroma [4].

Malignant plasma B cells characteristic of multiple myeloma (MM) are also believed to rely heavily on their interactions with elements of the surrounding microenvironment, including osteoclasts, osteoblasts, endothelial cells, macrophages and bone marrow stromal cells (BMSCs) [5]. Direct adhesive interactions between BMSCs and myeloma cells, as well as BMSC secretion of cytokines, chemokines, growth factors and other components, contribute to a symbiotic cycle which maintains a myeloma-promoting microenvironment and allows the tumor cells to thrive. These bidirectional interactions disrupt the normal hematopoietic process, induce osteolytic destruction of the bone, and contribute to resistance to conventional therapies [5]. For example, BMSC production of matrix proteins and factors such as fibronectin [6], insulin-like growth factor-1 (IGF-1) [7], stromal derived factor 1 alpha (SDF-1) [8], tumor necrosis factor alpha (TNF-α), B cell activating factor family (BAFF), and a proliferation inducing ligand (APRIL) [5] have all been shown to promote MM cell proliferation and resistance to conventional chemotherapeutic agents. These discoveries have contributed to the development of several recently approved targeted therapeutics, such as the proteasome inhibitor bortezomib, and lenalidomide, that are believed to exert their anti-MM effects at least in part by disrupting the interactions between MM cells and the other cells in tumor microenvironment [5,9].

MM is a complex and heterogeneous disease with respect to clinical manifestations and drug responses. Cytogenetic analyses and gene expression profiling studies further demonstrated the heterogeneity of MM cells in different patients [10-14]. It remains unclear how much of this complexity is due to differences in MM-BMSC interactions *in vivo*. Several studies comparing a small number of BMSCs from MM patients versus healthy donors suggested that these non-malignant support cells were not significantly different [15-17]. Accordingly, it is generally considered that BMSCs affect MM cells similarly, regardless of their specific sources. However, analyses of a more extensive sampling of MM and normal BMSCs exposed some important functional and phenotypic distinctions of MM BMSCs [18-23]. In particular, Wallace et al compared BMSCs from 8 MM patients and 9 normal healthy donors and found increased expression of both IL-1β and TNF-α by MM BMSCs [21]. Arnulf and colleagues analyzed BMSCs from 56 MM patients and 13 normal healthy donors and found that BMSCs from MM patients not only secreted higher levels of IL-6 than their normal counterparts, but also displayed an impaired ability to inhibit T cell proliferation in functional studies [18]. MM BMSCs have also been described to differentially express 145 distinct genes, about half of which are involved in tumor-stroma cross-talk, induce higher proliferation of a MM cell line, and are impaired in their ability to differentiate into osteoblasts [24]. Garayoa and colleagues recently analyzed BMSCs from 21 MM patients and 12 normal bone marrows for the presence of unbalanced genomic alterations. They found that while all 12 normal BMSCs were free from genomic alterations, BMSCs from 7 of the 21 MM patients displayed large gains or losses, indicating that these cells are also often distinct from their normal counterparts on a global genetic level [25]. Nonetheless, our understanding of the functional distinctions between BMSCs of diseased and non-diseased bone marrow is still incomplete.

The Rel/NF-κB family of transcription factors has been shown to play a pivotal role in the pathogenesis of many human cancers, including MM [12,26,27]. NF-κB is constitutively active to variable levels in primary and established MM cells, and has been shown to further increase in activity in response to a number of factors found in the bone marrow microenvironment [10,11,28,29]. For example, adhesion of myeloma cell lines to fibronectin results in increased NF-κB DNA-binding and correlates with induction of NF-κB-regulated genes [6]. IGF-1, which is secreted into the bone marrow milieu, induces NF-κB activity in MM cells, and inhibition of IGF-1 binding to its receptor induces apoptosis of MM cells [7]. Similarly, SDF-1 induces transient NF-κB DNA-binding activity in some primary MM cells [8]. Other well-known NF-κB inducers including TNF-α, IL-1β, BAFF, and APRIL are also present in the bone marrow [5].

NF-κB activation typically relies on two major pathways, canonical and noncanonical, involving degradation and processing of the inhibitor IκB and the precursor p100, respectively, via the ubiquitin-dependent proteasome pathway [30]. As such, both constitutive and inducible NF-κB activity in MM cells is generally believed to be highly sensitive to inhibition by the proteasome inhibitor bortezomib. This NF-κB inhibitory activity of bortezomib has been largely implicated in the clinical efficacy of this drug against MM and another B cell malignancy, Mantle Cell Lymphoma (MCL) [31]. In contrast to this view, we and others have recently shown that constitutive activity in primary MM cells is largely resistant to bortezomib [32,33]. Similarly, we also found that constitutive NF-κB activity in many primary MCL samples also showed bortezomib-resistance [34]. These studies indicated that primary MM and MCL, and possibly other cancer cell types, may behave differently than expected from cell line-based studies. Since NF-κB activity in MM is also dependent on the bone marrow milieu, whether NF-κB activation in primary MM cells through many of these microenvironment interactions is effectively prevented by bortezomib still remains an open question.

We previously showed that BMSCs generated from a few MM patients induced NF-κB activity in some primary and established MM cells. This activation is largely resistant to bortezomib and confers resistance to bortezomib-induced apoptosis [33]. In the present study, we expanded this analysis by examining BMSCs derived from marrows of a cohort of MM patients and those without MM pathology. Collectively, we found that MM-associated BMSCs are functionally different from non-MM BMSCs in their ability to induce bortezomib-resistant NF-κB activity in MM cells.

## Results

### BMSCs from MM patients are functionally distinct in their ability to activate NF-κB in MM cells

**Figure 1:**
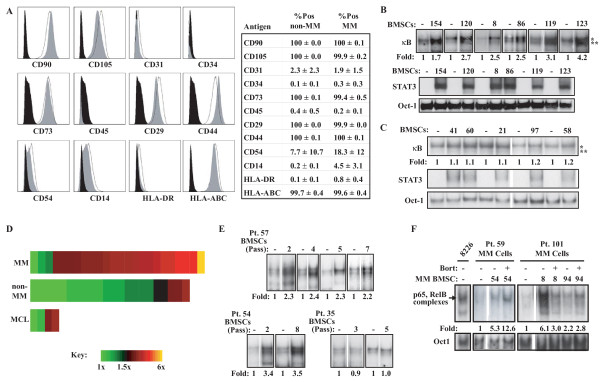
**BMSCs from MM patients selectively activate NF-κB in RPMI8226 cells and primary MM cells**. (A) Flow cytometric analysis comparing expression of cell-surface markers on MM-patient derived (black dashed-line) and normal bone marrow-derived (grey filled curves) BMSCs (isotype controls solid black). Curves shown are representative of eight BMSCs analyzed (four MM and four normal BMSC). The mean percentage positive cells with standard deviations are presented in the table. (B) EMSA analysis of NF-κB ("κB"), STAT3 ("STAT3") and Oct-1 ("Oct-1") DNA-binding in RPMI8226 cells cultured either alone or with BMSCs from the indicated MM patients for 24 hours. The two major NF-κB dimers active in RPMI8226 cells (p65-containing: one asterisk; RelB-containing: two asterisks) are labeled, and fold-change in NF-κB DNA-binding as measured by phosphoimage quantification is labeled below the κB gel. (C) Similar EMSA analysis of RPMI8226 cells cultured alone or with BMSCs from the indicated non-MM patients for 24 hours. (D) Fold-change in NF-κB activity in RPMI8226 cells induced by various MM-BMSCs ("MM") and non-MM BMSCs ("non-MM" and "MCL"), applied to a linear color gradient representing the range displayed as "Key." (E) NF-κB EMSA of RPMI8226 cells cultured alone or with BMSCs from the indicated patients, that had been cultured for the indicated number of passages. (F) Primary CD138^+ ^cells from the indicated patients were cultured alone or with BMSCs derived from the indicated MM patients, and then subjected to EMSA analysis with κB DNA-probe, or Oct-1 DNA-probe as a control. The main NF-κB dimers are labeled as compared to constitutive DNA-binding activity in RPMI8226 cells ("8226"). 100 nM bortezomib was added for the last four hours of co-culture where indicated ("Bort").

Because we have previously found that co-culture with MM BMSCs can activate NF-κB in the RPMI8226 MM cell line and a few primary MM samples in a manner that is largely resistant to bortezomib [33], we wanted to know (i) how variable this response is among BMSCs derived from different MM patients, and (ii) whether BMSCs derived from normal marrow possess similar NF-κB-inducing activities. To this end, we derived primary BMSC cultures from 25 patients with diagnosed MM, and from 21 normal marrows. Comparative analysis of cell surface marker expression characteristic of BMSCs showed few statistically significant differences (Figure 1A). Confirming previous reports, we found that CD54, also known as inter-cellular adhesion molecule-1 (ICAM-1), was heterogeneously expressed on the surface of MM-BMSCs, but very little on non-MM BMSCs [20]. Expression of other markers was similar to that previously reported in the literature. We were then interested in the potential functional differences, and first employed RPMI8226 and U266 established MM cell lines to measure the differential effects of MM or non-MM BMSCs on NF-κB activation. Confirming our previous data [33], when co-cultured with BMSCs from MM patients NF-κB activity in RPMI8226 was enhanced by several fold (Figure 1B). We observed similar results with the U266 cell line (data not shown). We chose to further pursue this phenomenon in the RPMI8226 cell line, because the constitutive level of NF-κB activity is so high in the U266 cell line, a 1.5-fold change in activity (like we saw with MM-BMSC coculture) though likely functionally significant, is difficult to measure robust changes in already high activity as measured by electrophoretic mobility shift assay. We have previously shown that our method of co-culture and cell-separation for analysis represents only NF-κB activity in the MM cells and that the measured NF-κB activity is not appreciably contaminated by BMSCs [33]. Surprisingly, BMSCs from non-MM patients largely did not affect NF-κB in these cells (Figure 1C). We observed striking heterogeneity in the degree of NF-κB activity induced by BMSCs from different MM patients, spanning from very little change to 6-fold increase in DNA-binding activity (Figure 1D, Additional File 1). The observed difference in fold-induction of NF-κB between the MM and non-MM groups was highly statistically significant (p = 0.001, Additional File 1). The two exceptions to this trend were BMSCs derived from patients with MCL, a B-cell malignancy that has many similarities to MM, including frequent involvement of the bone marrow [33]. Omission of the four MCL patients from the analysis revealed an even stronger difference statistically. Furthermore, the degree to which BMSCs from a given patient enhanced NF-κB activity in MM cells remained extremely stable within up to 8 passages in culture after removal from the patient (Figure 1E). This suggests that the specific ability of MM BMSCs to functionally affect MM cells is an inherent property of the BMSCs themselves and is maintained during culturing. As a positive control to demonstrate a general function of BMSCs, all of them, regardless of MM or non-MM source, were capable of robustly activating STAT3 DNA-binding activity in RPMI8226 cells ([28,35], Figure 1B and 1C), likely mediated by the significant levels of IL-6 secreted by both types of BMSCs (see below).

Co-culture experiments with primary MM cells from one patient and BMSCs from another patient assumedly introduces unknown variables. Nevertheless, we were interested to see if this differential ability of MM-derived BMSCs to activate NF-κB activity was also true in primary MM cells. To control for the variability in constitutive NF-κB activity between patients, the change in NF-κB activity after co-culture was normalized to the level of constitutive activity in the MM cells from the same patient, and all values were normalized to Oct-1 DNA-binding. These analyses demonstrated that BMSCs often activated NF-κB in primary MM (Figure 1F) but those from normal marrow frequently did not (Additional File 2). However, the heterogeneity of inter-patient experiments (i.e., the sources of MM cells and BMSCs coming from different patients) or small sample size failed to show statistical significance for the observed differences. As previously shown [32,33], bortezomib treatment often caused paradoxical increases in NF-κB activity in primary MM cells, showing that bortezomib-induced NF-κB activation, rather than inhibition, is a more common phenomenon, even in the presence of MM BMSCs (Figures 1F).

**Figure 2:**
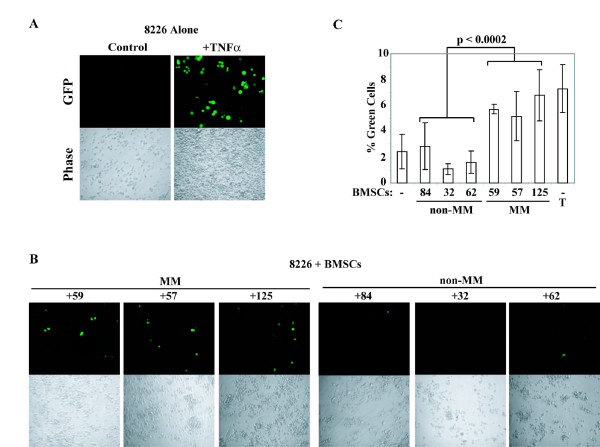
**BMSCs from MM patients specifically induce expression of a κB-dependent GFP reporter gene**. (A) Representative 20× image of RPMI8226 cells after transient transfection with a κB-GFP reporter construct that were cultured alone or treated with 1 ng/mL of TNF-α for 24 hours, visualized either through a GFP filter ("GFP"), or phase contrast ("Phase"). (B) Similar representative images of RPMI8226 cells transiently expressing the κB-GFP reporter that were cultured either alone or with BMSCs from the indicated MM or non-MM patients for 24 hours. (C) Quantification of the percentage of GFP-expressing cells ("% Green Cells") over total live cells with n = 3 for each co-culture condition or TNF-α treatment ("T"). Standard deviation is indicated by error bars and the p-value derived from statistical comparison of GFP expression induced by co-culture with non-MM versus MM BMSCs.

To corroborate biochemical data based on EMSA analysis to a more functional readout, we next examined the induction of a κB-dependent GFP reporter in RPMI8226 cells in the presence of BMSCs. The κB-GFP reporter construct was transiently transfected into RPMI8226 cells which were then placed on BMSCs, or exposed to TNFα as a positive control. We observed induction of κB-dependent GFP activity similar to that seen with TNFα treatment specifically by MM BMSCs, and not normal BMSCs (Figure 2A and 2B). The observed differences in the percentage of GFP-expressing cells was highly statistically significant (p = 0.001, Figure 2C). These studies revealed the previously unappreciated ability of MM BMSCs, but not non-MM BMSCs, to induce NF-κB activity in MM cells.

### MM BMSC-induced NF-κB activity is mediated by a polypeptide(s) secreted by BMSCs

**Figure 3:**
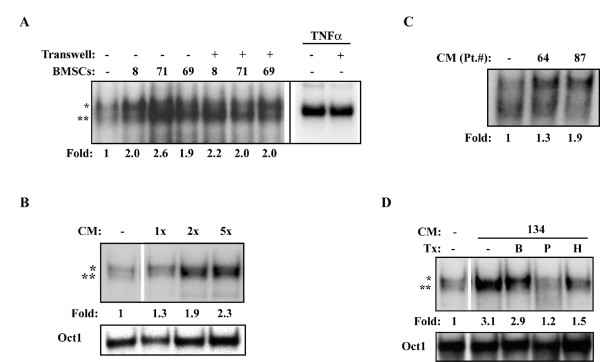
**MM BMSC-induced NF-κB activity is mediated by a secreted peptide factor**.(A) EMSA of NF-κB activity in RPMI8226 cells cultured alone or with BMSCs from the indicated MM patients, either directly, or physically separated by a Transwell insert ("Transwell"). 10 ng/mL TNF-α was placed either directly in RPMI8226 culture or below the Transwell insert as a positive control for membrane permeability. The black line indicates a different exposure time for the same gel for TNF-α-treated samples in order to better visualize the very different levels of binding activity. (B) EMSA of NF-κB and Oct-1 DNA-binding activity in RPMI8226 cells treated with 1× conditioned media (CM) or the same CM concentrated 2-fold (2×) or 5-fold (5×) for 24 hours. (C) EMSA of NF-κB activity in primary MM cells treated with CM derived from BMSCs of the indicated MM patients. (D) EMSA of NF-κB and Oct-1 DNA-binding activity in RPMI8226 cells treated with CM that had been previously treated with proteinase K ("P"), or heat ("H") where indicated. RPMI8226 cells were treated with CM from Pt. 134, and 100 nM bortezomib was added for the last 4 hours of culture where indicated ("B"). NF-κB dimers are labeled as before.

To gain insight into the nature of the MM BMSCs-derived factor(s) capable of inducing bortezomib-resistant NF-κB activity in MM cells, we next asked whether direct contact between RPMI8226 cells and BMSCs was necessary. We employed semi-permeable Transwell inserts to disrupt direct cell-cell contact. These inserts have 2 μM pores which prevent migration of cells, but allow diffusion of most small molecules, such as cytokines and growth factors. We found that a transwell insert did not significantly inhibit NF-κB activation in RPMI8226 cells induced by BMSCs or the positive control TNFα (Figure 3A). This result demonstrated that physical contact between MM cells and BMSCs is not necessary for NF-κB activation and that a soluble secreted factor(s) from BMSCs was mediating NF-κB activation. This notion was further corroborated by our observation that CM from MM-BMSCs was also able to activate NF-κB in RPMI8226 cells and primary MM cells (Figures 3B and 3C, respectively), and concentration of the CM by size-exclusion with a cut-off at 3 kDa resulted in further induced NF-κB activity (Figure 3B). Finally, the ability of BMSC CM to activate NF-κB activity in MM cells was abrogated by pre-treatment of the CM with proteinase K and by heating (Figure 3D), but not by DNase or RNase (not shown). Importantly, the soluble factor(s) present in CM from MM-BMSCs induced NF-κB activity via a largely bortezomib resistant mechanism (Figure 3D). These results indicate that BMSC-induced NF-κB activity in MM cells is mediated by a soluble secreted factor that is proteinaceous in nature and greater than 3 kDa.

A number of BMSC-produced protein factors have been shown to be important in MM pathogenesis, some of which can activate NF-κB in MM cells. Therefore, we wanted to know whether a factor (or factors) well-known to be produced by MM BMSCs was mediating bortezomib-resistant NF-κB activation in MM cells. To this end, a neutralizing antibody against TNFα, which was capable of blocking NF-κB activity induced by recombinant TNF-α in MM cells, did not block BMSC-induced NF-κB activity (Additional File 3A). This is consistent with the observation that TNF-α-induced canonical NF-κB activation is highly sensitive to inhibition by bortezomib in contrast to the factor that we were aiming to identify. SDF-1α (CXCL12), a factor that is believed to contribute to the maintenance of MM cells in the bone marrow, as well as to drug resistance and proliferation of the tumor cells [8], did not induce NF-κB activity in RPMI8226 cells (Additional File 3B). Additionally, a neutralizing antibody against SDF-1α also did not block BMSC-induced NF-κB DNA-binding activity in these cells (Additional File 3B). Similarly, a neutralizing antibody against IGF-1 receptor, whose ligation by IGF-1 has been shown to activate NF-κB in certain MM samples [29], had no effect on BMSC-induced NF-κB activity in RPMI8226 cells (Additional File 3B). Moreover, IL-6, another important factor in MM growth and survival, which is secreted at very high levels by BMSCs [12], also did not affect NF-κB activation in RPMI8226 cells (Additional File 3C).

BAFF and APRIL have been shown to be important in MM pathogenesis and can stimulate noncanonical NF-κB activation; however, we found that neither the levels of BAFF and APRIL mRNA or protein secreted from BMSCs correlated with their ability to induce NF-κB (Additional File 3D). In fact, APRIL protein levels were statistically significantly higher in the normal BMSC group (Additional File 3D). Collectively, these data indicate that the factor produced by MM BMSCs that is responsible for bortezomib-resistant NF-κB activation is likely distinct from these well-known BMSC products.

### IL-8 is a permissive factor for NF-κB activation in MM cells induced by some MM-BMSCs

**Figure 4:**
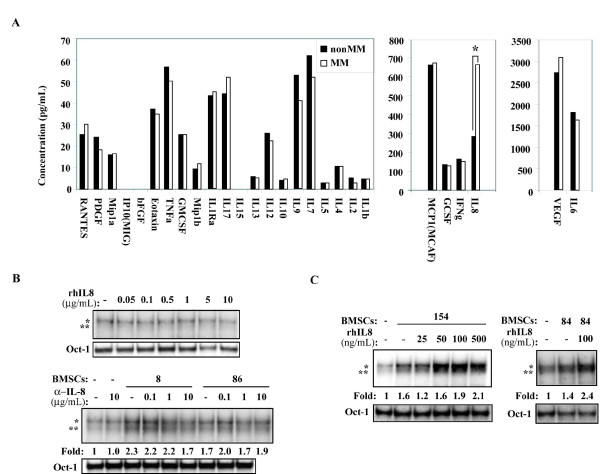
**IL-8 is differentially expressed by MM BMSCs and can potentiate BMSC-induced NF-κB activity in RPMI8226 cells**. (A) Median levels of 27 cytokines measured by cytokine array analysis in CM from 17 MM BMSCs (white bars) and 13 non-MM BMSCs (black bars). The scale is in pg/mL, and cytokines are grouped by relative concentration. Asterisk above IL-8 indicates a statistically significant difference between the groups determined by Mann-Whitney test. (B) NF-κB EMSA analysis of RPMI8226 cells treated with increasing doses of human recombinant IL-8 (top panel), and cultured alone or with BMSCs from the indicated patients with increasing amounts of anti-IL-8 neutralizing antibody as indicated (bottom panel). (C) EMSA analysis of NF-κB DNA-binding activity in RPMI8226 cells cultured alone or with BMSCs from the indicated patients with various concentrations of recombinant human IL-8. Oct-1 controls, NF-κB dimers, and fold-change in NF-κB activity was quantified and labeled as before.

To gain further insight into the nature of the BMSC-secreted factor capable of inducing bortezomib-resistant NF-κB activity, CM was collected from 17 MM-BMSCs and 13 non-MM BMSCs and a cytokine array was used to simultaneously measure protein levels of 27 human cytokines in each sample (Additional File 4). Median values for the MM and non-MM BMSCs are displayed for each of the 27 cytokines, grouped by relative range (Figure 4A). These cytokine array data showed that IL-8 was the only cytokine produced at statistically significantly higher levels by MM-BMSCs (p = 0.05, Figure 4A). Therefore, we next asked if IL-8 was involved in BMSC-induced NF-κB activity in RPMI8226 cells. IL-8 has not been previously shown to cause NF-κB activation in MM cells. Concordantly, when we exposed RPMI8226 cells to recombinant human IL-8 at various concentrations, we did not observe any evidence of NF-κB activation (Figure 4B, top panel), suggesting that IL-8 alone is insufficient to cause NF-κB activation. In contrast, use of anti-IL-8 neutralizing antibody in the context of co-culture experiments resulted in partial inhibition of NF-κB activity induced by some patient BMSCs (Figure 4B, bottom panel), suggesting that IL-8 may be necessary for NF-κB activation induced by certain BMSCs. These data indicate that IL-8 collaborates with another factor(s) produced by BMSCs to induce NF-κB activity in MM cells. Consistent with this, exogenous IL-8, although unable to directly cause NF-κB activity, was capable of potentiating NF-κB activation by some BMSCs (Figure 4C). Collectively, our data show that IL-8 is a novel factor that can potentiate NF-κB activation induced by certain MM BMSCs.

**Figure 5:**
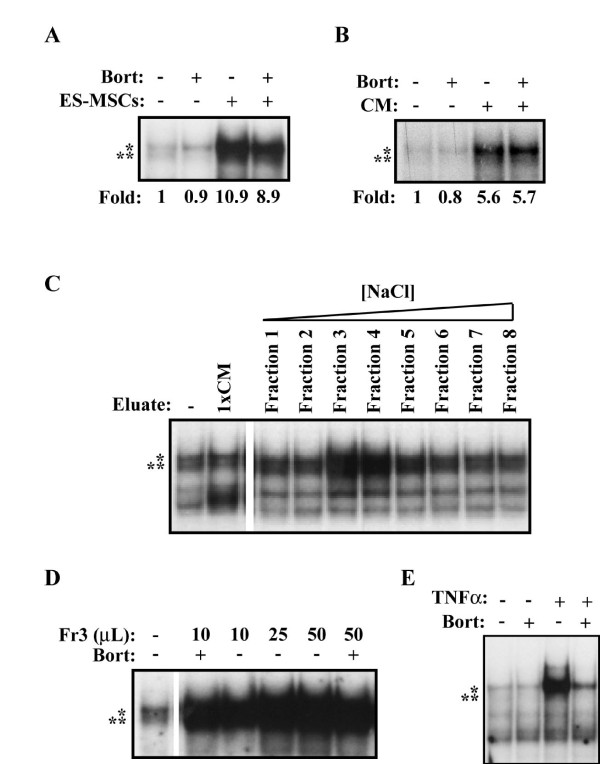
**Partial purification of NF-κB-inducing factor derived from MSCs**. (A) EMSA analysis of RPMI8226 cells cultured alone or with H1-ESC-MSCs (ES-MSCs) for 24 hours with or without 4-hour treatment of 100 nM bortezomib (Bort). (B) EMSA analysis of RPMI8226 cells cultured alone or with CM from H1-ESC-MSCs (CM) for 24 hours with or without 4-hour treatment with 100 nM bortezomib (Bort). NF-κB dimers and fold-change in DNA-binding activity are displayed as before. (C) EMSA analysis of RPMI8226 cells untreated (OT) or treated with 1× CM or sequential fractions eluted from anion-exchange column with increasing NaCl concentration gradient (Fraction 1-8). (D) EMSA analysis of RPMI8226 cells untreated or treated with increasing aliquots of an activity-containing fraction eluted from the anion-exchange column (Fr3), with or without treatment with 100 nM bortezomib (Bort). (E) EMSA analysis of RPMI8226 cells treated with 100 nM bortezomib (bort), 10 ng/mL rhTNF-α (TNF-α) or both.

We next wanted to further characterize the nature of the unknown factor (or factors) capable of inducing bortezomib-resistant NF-κB activity in MM cells. However, limited cell-culture expansion of MM patient-derived BMSCs precluded extensive biochemical purification of the CM. Furthermore, the factor(s) responsible for NF-κB-inducing activity was found to be labile and lost with freeze-thaw cycles and prolonged incubation at 4°C (data not shown), prohibiting cumulative collection of CM. In a set of pilot studies we investigated the effect of mesenchymal stem cells (MSCs) derived from human embryonic stem cells [36,37] on MM cells. These ESC-derived MSCs express cell surface markers and possess multilineage differentiation potential similar to normal bone marrow MSCs [36]. Analysis of a H1 ESC-derived MSCs showed these cells produced a factor (or factors) capable of robustly inducing NF-κB activity in MM cells in a manner resistant to bortezomib treatment (Figure 5A and 5B). The NF-κB inducing factor produced by these MSCs was also found to be similarly labile as that from MM-BMSCs and could be enriched by cation affinity ion-exchange chromatography (Figure 5C). This purification demonstrated that the responsible factor is acidic at neutral pH. Importantly, NF-κB activity induced by these fractions was highly resistant to bortezomib (Figure 5D) even though the same high concentration of bortezomib could effectively prevent NF-κB activation induced by TNF-α treatment (Figure 5E). These studies show that these human ESC-derived MSCs could be a good source for purifying a novel acidic factor that induces NF-κB activity in a bortezomib-resistant fashion. Further studies are currently ongoing in our laboratory.

## Discussion

The tumor microenvironment is thought to participate in many aspects of human cancer. In MM, BMSCs and the factors and matrix that they produce are important in tumor cell proliferation and drug resistance. NF-κB has been implicated in this dynamic MM cell-stromal communication and several components along the NF-κB signaling pathway are frequent targets of genetic modification leading to constitutive activity in MM cells [10,11], further supporting the important role that this signaling system plays in MM pathogenesis. In addition, NF-κB activation in non-malignant BMSCs leads to increased expression of cytokines and growth factors such as IL-6, TNF-α, and VEGF [5], which can further increase NF-κB activity in MM cells. This NF-κB activation in both MM cells and BMSCs is proposed to create positive feedback loops to promote symbiotic interactions and survival of MM cells in the bone marrow. However, BMSCs from MM patients are generally considered to be indistinct from those in a normal healthy individual, with the potential consequence of overlooking novel drug targets and/or markers of disease that are features specifically of MM-BMSCs.

We have previously shown that BMSCs from MM patients variably activate NF-κB in several MM cell lines and primary MM cells in a manner that is resistant to bortezomib. In the present study we addressed how BMSCs from MM patients and normal marrow differentially affect NF-κB activity in MM cells. We employed the MM cell line RPMI8226 to examine the functional difference between a large cohort of patient-derived MM and non-MM BMSCs. MM-BMSCs were found to cause NF-κB activation in these cells with a strong statistical significance over those from morphologically normal marrow. The NF-κB-inducing activity of the BMSC cultures did not change over 8 passages in culture. This indicated that the phenotype of BMSCs with respect to this functionality is quite stable in culture and therefore reflects an intrinsic property, rather than dependence on continual interaction with primary MM cells. Thus, our study represents the first instance in which MM-BMSCs were shown to possess an intrinsic and stable difference from those of non-myeloma marrow with respect to activation of NF-κB.

The more consistent expression of CD54 (ICAM-1) on MM BMSCs is of unknown significance. Noborio-Hatano and colleagues showed that downregulation of CD54 expression *on MM cells *using short-hairpin RNA decreased cell-adhesion mediated drug resistance of MM cells to bortezomib [38]. It is possible, in a similar sense, that disrupting the ICAM-1 (on BMSCs) -LFA-4 or -Mac-1 (on MM cells) interaction could also lead to increased susceptibility of MM cells to bortezomib. More extensive studies are required to test this hypothesis.

To extend our findings with MM cell lines, we also analyzed the effect of various BMSCs on primary MM cells isolated from different patients and found a trend analogous to RPMI8226 cells. These primary cell studies were complicated, however, by multiple confounders. First, constitutive levels of NF-κB activity in primary MM cells are quite variable from patient to patient. Thus, if MM cells from a given patient have nearly undetectable NF-κB activity, any increase in DNA-binding will manifest in a large fold-change. In contrast, a small increase in MM cells that already harbor a very high basal activity might have a significant functional impact even though the fold-change may seem inconsequential when represented graphically. Second, the current experimental set up necessitates that primary MM cells from one patient are placed on BMSCs derived from another patient, i.e., "*inter*-patient" analysis. This design introduces an unknown level of complexity, which can only be avoided by performing "*intra*-patient" experiments. Currently, intra-patient experiments require cryopreservation of primary MM cells during the derivation of BMSCs from the same patient, a process which takes weeks beyond the viability of primary MM cells in culture. Finally, the unknown effect of chemotherapeutic agents on the microenvironment, and the heterogeneity of the biopsy material, due to factors such as location and timing during treatment on MM and BMSC sampling introduce additional confounding complexities to such analyses. Therefore, one of the major future challenges is to develop strategies to overcome such logistical issues and develop *intra*-patient experimental strategies to further reveal mechanisms relevant to individual MM patients at different points in their treatment course. We also were not able to correlate our findings in any way to patient characteristics, disease stage, or response to therapy because the samples used in these studies were collected under an IRB-exempt protocol. Future studies will be performed with samples that have accompanying patient information in order to obtain maximal insight about MM pathology.

NF-κB is thought to have a significant role in MM pathogenesis, and we have shown that bortezomib-resistant NF-κB activity correlates with cellular resistance to bortezomib *in vitro *[33]. Therefore, identification of a novel factor(s) produced by BMSCs that activates this transcription factor could potentially provide a novel biomarker or therapeutic target. Although key components found in the marrow, such as TNF-α, SDF-1, IGF-1, IL-6, BAFF, and APRIL, are involved in MM pathogenesis, we did not find evidence that these factors were necessary for BMSC-induced, bortezomib-resistant NF-κB activity in MM cells. Some of these (IL-1β, TNF-α, IL-6, and IL-8) have been described to be produced at significantly higher levels by MM BMSCs than their normal counterparts [18,20-22]; however, cytokine array analysis of our BMSC cohorts detected a statistically significant difference of only IL-8. A recent study comparing production of IL-8 by BMSCs derived from patients with monoclonal gammopathy of undetermined significance (MGUS), an asymptomatic disease of increased immunoglobulin secretion by plasma cells, smoldering MM, or active MM, found that IL-8 levels correlated positively with disease state [39]. In addition to its pro-angiogenic effects in MM [40], our data suggest the possibility that IL-8 is also a contributing factor to increase NF-κB activity in some patients. Although our cytokine array data suggest that the majority of soluble IL-8 is being secreted by BMSCs, MM cells themselves can secrete IL-8 and may contribute to this phenomenon in an autocrine fashion.

MM-BMSCs are producing an additional factor (or factors) that together results in a strong signal to activate NF-κB in MM cells. This does not require direct contact between BMSCs and MM cells. The responsible factor(s) is sensitive to treatment with proteinase K and heat, and can be concentrated with a 3 kDa cut-off, suggesting that the factor(s) is a polypeptide of greater than 3 kDa. Because this BMSC-secreted factor causes NF-κB activation in MM cells in a manner resistant to bortezomib [33], this would also represent the first known factor to induce proteasome inhibitor resistant (PIR) NF-κB activation in MM cells. The signal transduction pathway leading to PIR NF-κB activation in B cells is not completely understood, although we have described several critical events, including the role of calcium and calmodulin [41-43]. Efforts to isolate and identify the responsible factor from MM patient BMSCs were limited by their heterogeneity and the limited amount of conditioned media obtained from these cells. Therefore, we employed an H1-ESC-derived MSC line which also produces a factor (or factors) that caused bortezomib-resistant NF-κB activity in MM cells for initial purification studies. We were able to enrich this factor by ion exchange chromatography. Further purification steps are required to distil and identify the responsible factor(s).

## Conclusions

Our findings and others' indicate that the BMSCs in patients with MM are functionally distinct from those in normal marrow, the implications of which are not fully understood. Because of the differentially ability of MM BMSCs to uniquely induce the bortezomib-resistant NF-κB pathway, we have reason to believe that further investigating into exactly how BMSCs differ in MM patients could lead to improved understanding of MM and therefore improved treatment of MM patients. Identification of the MM BMSC factor would not only permit further dissection of this putatively novel NF-κB signaling pathway in MM, but also could provide useful markers for drug resistance and/or potential drug targets to be used in conjunction with targeted therapies such as bortezomib and lenalidomide. Because we also observed a similar NF-κB-inducing activity in some BMSCs derived from MCL patients and bortezomib-resistant activity is also prevalent in primary MCL samples [34], such a factor could also play a role in promoting MCL pathogenesis. Thus, our significant future goal is to molecularly identify this factor (or factors) and examine its role in MM and MCL pathologies.

## Methods

### Cell lines, antibodies, and chemicals

The RPMI8226 cell line was cultured as previously described [33]. Bortezomib was commercially obtained from Millennium Pharmaceuticals, Inc. (Cambridge, MA) for experimental purposes, proteinase K was from Promega (Fitchburg, WI), and human recombinant TNF-α from Calbiochem (San Diego, CA). Recombinant human SDF-1, IL-8, IL-6, anti-SDF-1 and anti-IL-8 neutralizing antibodies were from R&D systems (Minneapolis, MN). Anti-IGF-1R neutralizing antibody was from Calbiochem (Darmstadt, Germany), and anti-TNF-α antibody from Chemicon International (Temecula, CA).

### Primary BMSCs and MM Cells

Primary human BMSCs were derived from de-identified fresh whole bone marrow aspirates, under the University of Wisconsin Institutional Review Board exemption protocol #M-2004-1315. MM patients had active disease at the time of sampling, and "non-MM" patients were determined to have morphologically normal marrow by UW pathologists. Because the protocol by nature precludes collection of any identifying information, the samples collected were only accompanied by diagnosis, and no information regarding disease stage, treatment history or treatment response. Mononuclear cells were isolated from aspirates as detailed in the Miltenyi MidiMACS bone marrow mononuclear cell protocol and as described previously [33]. Mononuclear cells isolated from MM patient bone marrow aspirates were positively sorted using anti-CD138 magnetic microbeads and the MidiMACS cell sorting system following manufacturer's protocol (Miltenyi Biotec, Auburn, CA) to >95% purity as determined by anti-CD138-PE staining and FACS analysis, and cultured in 37°C/5%CO_2 _incubators in BMSC media.

### Human ESC-derived MSCs

MSCs were derived from the H1 human embryonic stem cell (ESC) line as previously described [36]. These cells were cultured in αMEM media (Invitrogen, Carlsbad, CA, USA) containing 20% fetal bovine serum, 0.1 mM nonessential amino acid (NEAA), and 2 mM L-glutamine (glu). For collection of CM, H1-ES-MSCs were cultured in phenol red-free αMEM media supplemented with only 0.1 mM NEAA and 2 mM glu.

### Co-culture, transwell, and conditioned media (CM) assays

6 × 10^4 ^BMSCs per well were plated in 6-well dishes and allowed to form an adherent monolayer over 24 hours. 10^6 ^RPMI8226 cells or 5 × 10^5 ^primary MM cells were plated over the monolayer for an additional 24 hours and then MM cells were mechanically detached from the stromal layer. The BMSC monolayer was undisturbed. Where appropriate, 100 nM bortezomib was added for the last 4 hours of co-culture. Neutralizing antibodies and/or recombinant proteins were added at the initiation of co-culture and, in the case of neutralizing antibodies, were pulsed every 6 hours for the duration of co-culture.

For transwell experiments, a monolayer of BMSCs was plated as described above, the Transwell insert (BD Biosciences, San Jose, CA) was placed over the BMSCs, and RPMI8226 cells placed within the insert.

BMSC CM was prepared by first washing pre-established BMSCs twice with 1× PBS then incubating them in serum free "BMSC media" for 48 hours. This media was clarified by centrifugation at 13,000 × g for 1 minute. CM was concentrated using Amicon Ultra Centrifugal Filter Devices with a 3 kDa molecular weight cut off according to manufacturer's instructions (Millipore, Billerica, MA), treated with 200 μg/mL proteinase K at 37°C for one hour followed by deactivation with 0.1 mM phenylmethylsulphonyl fluoride, or heated to 100°C for 30 minutes and then centrifuged for one minute at 13,000 × g.

### Partial enrichment of human ESC-derived MSC-induced factor

Conditioned media was prepared by culturing a confluent monolayer of human ESC-MSCs between 4 and 10 passages, washing the monolayer 2× with 1× PBS, and culturing the cells in serum-free, phenol-red free αMEM media with L-glutamine, penicillin/streptomycin, sodium bicarbonate and non-essential amino acids. After 48-72 hours, this conditioned media was aspirated and centrifuged at 10,000 × g for 5 minutes to remove cellular debris. A portion of the albumin (likely human, produced by the MSCs) was removed using Affigel Blue (Bio-Rad) resin at 1:20 dilution by stirring at 4 degrees for one hour, which did not appreciably affect NF-κB-inducing activity (data not shown). The conditioned media was again centrifuged at 10,000 × g for 5 minutes and vacuum filtered over a PVDF membrane with 2 μm pores to remove resin and air bubbles. After equilibration at 4 degrees, the conditioned media was run onto a 1.0 mL Mono Q 5/50 GL (GE Life Sciences) anion exchange column (IEX) at ~1.5 MPa with 20 mM TRIS-HCl, pH 8.0 starting buffer to bind cationic molecules, using a benchtop ÄKTA FPLC (Amersham) at 4°C. Bound material was then eluted using a linear salt gradient from 0-0.5 M NaCl over 30 column volumes, and 1.0 mL fractions were collected and assayed for NF-κB-inducing activity by treating RPMI8226 cells with fractions at a 1:50 dilution in serum free media for 24 hours and performing EMSA.

### Electrophoretic mobility shift assays (EMSA)

EMSA for cell lines were performed as described previously [44], using double strand DNA probes with the following sequences: κB: 5'-TCAACAGAGGGACTCCGAGAGGCC-3', Oct-1 [41], and STAT3: 5'-CGGGAGGGATTTACGGGAAATGCTA-3'. For primary MM cells, miniaturized EMSA ("mini-EMSA") was performed as previously described [33]. Briefly, approximately 100,000 CD138^+ ^cells were lysed in 5 μL of Totex extraction buffer and 1.5 μL of this extract were used for each reaction in NF-κB and Oct-1 DNA-binding assays. NF-κB binding was normalized to Oct-1 binding to control for equal protein loading.

### GFP-reporter assay

κB-dependent reporter assay was performed as described previously [33].

### Quantitative real-time reverse transcriptase-polymerase chain reaction (qRT-PCR) and enzyme-linked immunosorbent assay (ELISA)

For BAFF and APRIL ELISA assays, CM was collected from BMSCs as described above, and frozen for later batch comparison. 50 μL aliquots of CM were thawed on ice, aliquoted in duplicate into a 96-well instant BAFF ELISA plate, or APRIL ELISA plate (Bender MedSystems, Burlingame, CA), and processed according to manufacturer's instructions.

Total RNA was collected from 10^5 ^BMSCs from each patient using Trizol reagent (Invitrogen, Carlsbad, CA), used to make cDNA, and qRT-PCR performed on a BioRad iQ5, with the following primers: BAFF: 5'-GGGAGCAGTCACGCCTTAC-3' (forward), 5'-CTGGGGAGGATGGAAACACAC-3' (reverse); APRIL: 5'-CTCTGCTGACCCAACAAACAG-3' (forward), 5'-TTTTCCGGGATCTCTCCCCAT-3' (reverse); GAPDH: 5'-GAAGGTCGGAGTCAACGGATTT-3' (forward), 5'-GAATTTGCCATGGGTGGAAT-3' (reverse).

### Cytokine Array

CM from BMSCs was prepared as described above and kept frozen at -80°C for later batch comparison. Aliquots of CM were thawed on ice and placed in duplicate into a 96-well dish, and concentrations of 27 human cytokines included in the Human 27-Plex Panel (Bio-Rad, Hercules, CA, USA) were measured in each well using the Luminex fluorescent bead-based multiplex cytokine assay system (Bio-Rad, Hercules, CA, USA) according to the manufacturer's instructions. Data were analyzed with the Bio-Plex Manager software program.

### Flow Cytometry

Antibodies used were anti-CD31 Allophycocyanin (APC), anti-CD45 Phycoerythrin (PE), anti-CD90 APC (eBioscience, San Diego, CA, USA), anti-CD 54 Fluorescein isothiocyanate (FITC), anti-CD105 APC, anti-HLA-ABC FITC (Invitrogen, Carlsbad, CA, USA), anti-CD14 FITC, anti-CD29 PE, anti-CD34 APC, anti-CD44 PE, anti-CD73 PE, anti-HLA-ABC FITC, anti-HLA-DR FITC (BD Pharmingen, San Diego, CA, USA), IgG2a FITC isotype control antibody, IgG1 PE isotype control antibody, IgG1 APC isotype control antibody (Miltenyi Biotech). For surface staining, cells were harvested by trypsinization for two minutes at 37°C. After centrifugation, supernatant was aspirated then antibodies were added and incubated for 30 min at 4°C in dark. Cells were then washed with PBS containing 0.5% BSA and 2 mM EDTA. 100,000 cells per sample were analyzed using a Accuri C6 flow cytometer (Accuri cytometers, Ann Arbor, MI, USA), and FlowJo software version 7.2.5 (Tree Star, Ashland, OR, USA). BMSCs from 4 MM patients and 4 normal marrow were analyzed.

### Statistical analysis

Fold-induction of NF-κB activity was determined by phosphoimage quantification of enhanced NF-κB activity over constitutive activity, which was assigned an arbitrary value of one. Observed differences between MM and non-MM BMSCs were assessed for statistical significance using either Student's t-test or Mann-Whitney test depending on whether the normality assumption for the distribution of the data was reasonable (GFP expression, BAFF and APRIL protein levels, and the differential expression of 27 cytokines) or not (fold change in NF-κB activity and BAFF and APRIL mRNA levels). All tests were performed as a two-sided test, and the observed significance level, i.e. p-value, less than 0.05 was considered to indicate statistical significance of the observed differences

## Competing interests

The authors declare that they have no competing interests.

## Authors' contributions

StM directed the experimental design, execution and analysis, drafted the manuscript, and prepared the figures. NS and CL provided access to clinical samples, contributed ideas to experimental design and editing of the manuscript. SO contributed to the experimental design and editing of the manuscript. GX and YS performed and analyzed cytokine array analysis. KK provided statistical oversight and contributed to editing the manuscript. PT generated the H1ESMSCs and provided her expertise with these cells as well as editing the manuscript. JK and PH performed and analyzed flow-cytometry analysis of BMSCs and edited the manuscript. ShM provided oversight and expertise for the experimental design, execution and analysis, editing the manuscript and figures. All authors have read and approved the final manuscript.

## Supplementary Material

Additional file 1 **Effect of MM-BMSCs on NF-κB activity in RPMI8226 cells**. Fold-change in NF-κB activity induced by BMSCs as a group of either MM, non-MM including MCL patients, non-MM excluding MCL patients, and MCL patients alone. Median fold change is labeled by horizontal lines, with p-values determined by Mann-Whitney analysis below. Click here for file

Additional file 2 **BMSCs from MM patients activate NF-κB in primary MM cells more than BMSCs from non-MM patients**. (A) Color heat map was generated with fold-change in NF-κB activity in primary MM cells induced by various MM-BMSCs ("MM") and non-MM BMSCs ("non-MM"), these numbers were applied to a linear color gradient representing the range displayed as "Key." (B) Fold-change in NF-κB activity induced in primary MM cells by BMSCs as a group of either MM or non-MM patients. Mean fold change is labeled by horizontal lines. Click here for file

Additional file 3 **MM BMSC-induced NF-κB activity is mediated by a factor that is distinct from many known BMSC products**. (A) NF-κB EMSA of RPMI8226 cells treated with TNFα and/or anti-TNFα neutralizing antibody (left panel), or cultured alone or with BMSCs with the addition of increasing concentrations of anti-TNFα neutralizing antibody as indicated (right panel). (B) NF-κB EMSA of RPMI8226 cells treated with increasing concentrations of recombinant human SDF-1α (top left panel) or RPMI8226 cells cultured alone or with BMSCs with the addition of anti-SDF-1α or anti-IGF-1R neutralizing antibodies where indicated (top right and bottom panels). (C) EMSA of NF-κB DNA-binding in RPMI8226 cells treated with increasing concentrations of recombinant IL-6 as indicated. NF-κB dimers for A-C are labeled as before. (D) Relative mRNA (left panels) and protein levels (pg/mL, right panels) measured by qRT-PCR and ELISA, respectively, of BAFF and APRIL expressed by BMSCs derived from the indicated MM and non-MM patients. Standard deviations are represented by error bars for qRT-PCR with n = 3. Click here for file

Additional file 4 **Range and standard deviations of MM BMSC secretion of many cytokines is greater than those of non-MM BMSCs**. Levels of cytokines measured in the cytokine array with conditioned media from each BMSC in the MM (open circles) and non-MM (closed circles) group. Cytokines for which the range and standard deviation was greater in the MM group than the non-MM group are indicated by single stars. Cytokines for which removal of a single outlier point in the non-MM group makes the range and standard deviation greater for the MM group are indicated with double stars. Click here for file
