# Supplementary figures and images for: Bone marrow stromal cells from multiple myeloma patients uniquely induce bortezomib resistant NF-κB activity in myeloma cells
Source: Mol Cancer. 2010 Jul 6;9:176. doi: 10.1186/1476-4598-9-176 (PMC3095250; doi:10.1186/1476-4598-9-176)

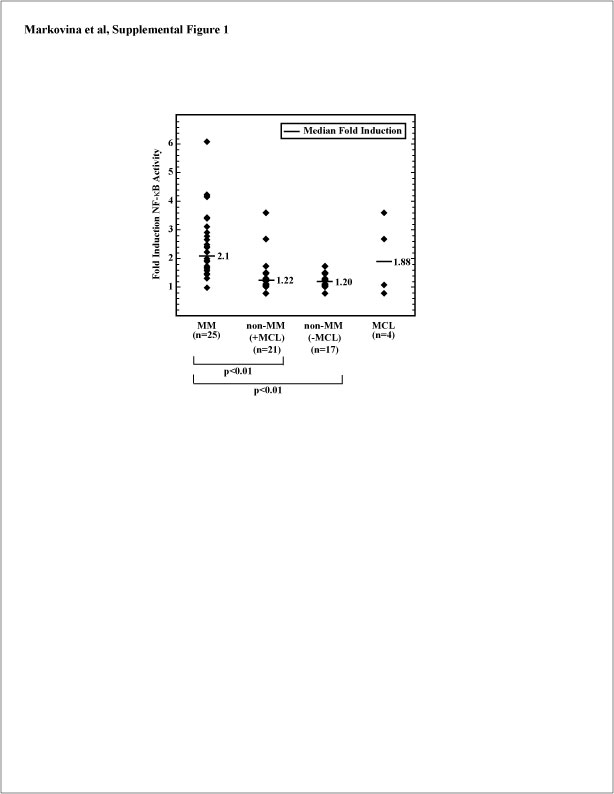

Supplement: Additional file 1 — Effect of MM-BMSCs on NF-κB activity in RPMI8226 cells. Fold-change in NF-κB activity induced by BMSCs as a group of either MM, non-MM including MCL patients, non-MM excluding MCL patients, and MCL patients alone. Median fold change is labeled by horizontal lines, with p-values determined by Mann-Whitney analysis below. [file 1476-4598-9-176-S1.JPG]

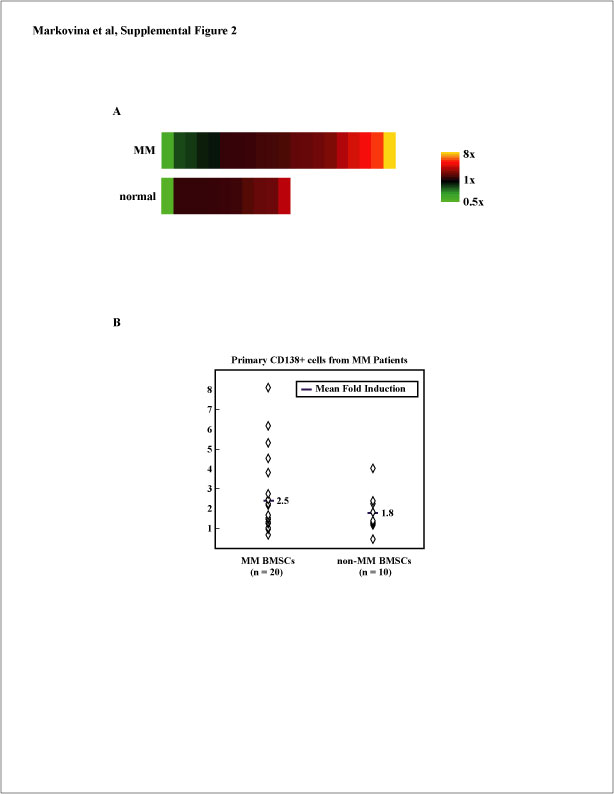

Supplement: Additional file 2 — BMSCs from MM patients activate NF-κB in primary MM cells more than BMSCs from non-MM patients. (A) Color heat map was generated with fold-change in NF-κB activity in primary MM cells induced by various MM-BMSCs ("MM") and non-MM BMSCs ("non-MM"), these numbers were applied to a linear color gradient representing the range displayed as "Key." (B) Fold-change in NF-κB activity induced in primary MM cells by BMSCs as a group of either MM or non-MM patients. Mean fold change is labeled by horizontal lines. [file 1476-4598-9-176-S2.JPG]

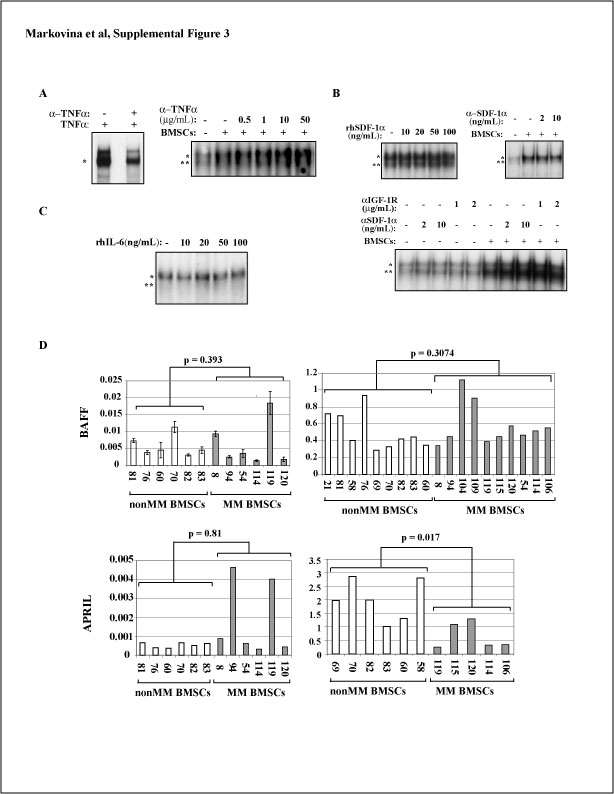

Supplement: Additional file 3 — MM BMSC-induced NF-κB activity is mediated by a factor that is distinct from many known BMSC products. (A) NF-κB EMSA of RPMI8226 cells treated with TNFα and/or anti-TNFα neutralizing antibody (left panel), or cultured alone or with BMSCs with the addition of increasing concentrations of anti-TNFα neutralizing antibody as indicated (right panel). (B) NF-κB EMSA of RPMI8226 cells treated with increasing concentrations of recombinant human SDF-1α (top left panel) or RPMI8226 cells cultured alone or with BMSCs with the addition of anti-SDF-1α or anti-IGF-1R neutralizing antibodies where indicated (top right and bottom panels). (C) EMSA of NF-κB DNA-binding in RPMI8226 cells treated with increasing concentrations of recombinant IL-6 as indicated. NF-κB dimers for A-C are labeled as before. (D) Relative mRNA (left panels) and protein levels (pg/mL, right panels) measured by qRT-PCR and ELISA, respectively, of BAFF and APRIL expressed by BMSCs derived from the indicated MM and non-MM patients. Standard deviations are represented by error bars for qRT-PCR with n = 3. [file 1476-4598-9-176-S3.JPG]

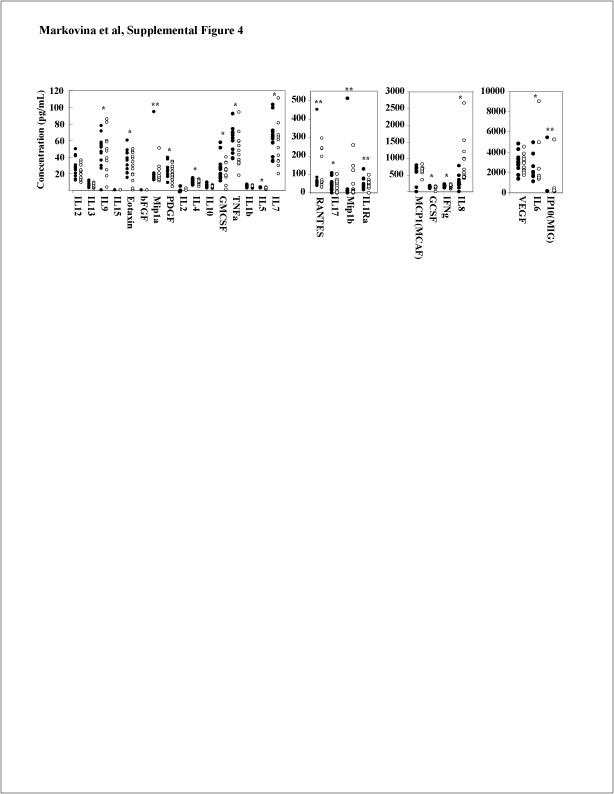

Supplement: Additional file 4 — Range and standard deviations of MM BMSC secretion of many cytokines is greater than those of non-MM BMSCs. Levels of cytokines measured in the cytokine array with conditioned media from each BMSC in the MM (open circles) and non-MM (closed circles) group. Cytokines for which the range and standard deviation was greater in the MM group than the non-MM group are indicated by single stars. Cytokines for which removal of a single outlier point in the non-MM group makes the range and standard deviation greater for the MM group are indicated with double stars. [file 1476-4598-9-176-S4.JPG]
